# Costo-efectividad de la prueba de secuenciación del gen *CFTR* para portadores asintomáticos en la población colombiana

**DOI:** 10.7705/biomedica.4816

**Published:** 2020-06-30

**Authors:** Ernesto Andrade, Jorge Díaz

**Affiliations:** 1 Instituto de Investigaciones Clínicas, Universidad Nacional de Colombia, Bogotá, D.C., Colombia Universidad Nacional de Colombia Instituto de Investigaciones Clínicas Universidad Nacional de Colombia BogotáD.C Colombia

**Keywords:** fibrosis quística/genética, tamización de portadores genéticos, asesoramiento genético, pruebas genéticas, evaluación de costo-efectividad, Cystic fibrosis/genetics, genetic carrier screening, genetic counseling, genetic testing, cost-effectiveness evaluation

## Abstract

**Introducción.:**

La fibrosis quística es una enfermedad genética de carácter autosómico recesivo clasificada como enfermedad huérfana de alto costo.

**Objetivo.:**

Determinar la razón de costo-efectividad de la prueba diagnóstica de secuenciación del gen *CFTR* para los portadores asintomáticos familiares en primer, segundo y tercer grados de consanguinidad.

**Materiales y métodos.:**

Se hizo una búsqueda sistemática sobre la evaluación de las características operativas de la prueba diagnóstica y los modelos de árbol de decisiones en estudios de costo-efectividad. Se elaboró un modelo de árbol de decisiones tomando como unidad de análisis la prevención de futuras concepciones. Los costos de la enfermedad se obtuvieron del reporte de alto costo del Ministerio de Salud de Colombia. Los costos de la prueba se obtuvieron de laboratorios nacionales. Se hizo un análisis de sensibilidad, determinístico y probabilístico, con la perspectiva del tercer pagador y horizonte a un año.

**Resultados.:**

Se obtuvo una razón incremental de *costo-efectividad* (RICE) de USD$ 5.051,10 por obtener 10,89 % más de probabilidades de evitar el nacimiento de un niño enfermo con fibrosis quística por pareja. Para los familiares de segundo y tercer grados, se encontró una RICE de USD$ 19.380,94 y USD$ 55.913,53, respectivamente, al aplicar el PIB per cápita. Esta tecnología fue costo-efectiva en 39 %, 61,18 % y 74,36 % para 1, 2 y 3 PIB per cápita en familiares de primer grado de consanguinidad.

**Conclusiones.:**

La prueba genética de detección de portadores del gen *CFTR* resultó costo-efectiva dependiendo del umbral de la disponibilidad de pagar, y de los supuestos y limitaciones establecidas en el modelo.

## Abstract (en)

**Introduction:**

Cystic fibrosis is an autosomal recessive genetic disease classified as a high- cost orphan disease.

**Objective:**

To determine the cost-effectiveness ratio of the diagnostic test for the *CFTR* gene-sequencing in asymptomatic family carriers in the first, second, and third degree of consanguinity.

**Materials and methods:**

We conducted a systematic search evaluating operative characteristics of the diagnostic test and decision-tree models in cost-effectiveness studies. A decision-tree model was elaborated taking prevention of future conceptions as a unit of analysis. We obtained the costs of the disease from the high-cost report of the *Ministerio de Salud y Protección Social*. The costs of the test were referenced by national laboratories.

We carried out a deterministic and probabilistic sensitivity analysis with a third-payer perspective and a one-year horizon.

**Results:**

An ICER of USD$ 5051.10 was obtained as the incremental cost for obtaining 10.89% more probability of avoiding the birth of a child with cystic fibrosis per screened couple. For family members in second and third degrees, the ICER was USD$ 19,380.94 and USD$ 55,913.53, respectively, evidenced when applying the GDP per capita. This technology was cost-effective in 39%, 61.18%, and 74.36% for 1, 2, and 3 GDP per capita in first degree of consanguinity relatives.

**Conclusions:**

The genetic test for the detection of *CFTR* gene carriers was cost-effective depending on the threshold of availability to pay and the assumptions and limitations established in the model.

La fibrosis quística es una enfermedad genética de carácter autosómico recesivo clasificada como enfermedad huérfana de alto costo. Es considerada la enfermedad genética más prevalente del grupo de las autosómicas recesivas, con una elevada incidencia mundial en caucásicos, aproximadamente de uno de cada 2.500 nacidos vivos ^1^.

La incidencia mundial de la fibrosis quística varía; en Europa, se presenta en uno de cada 2.000 a 3.000 nacidos; en África, en uno de cada 7.056; en Norteamérica, en uno de cada 3.500; en Latinoamérica varía entre uno de cada 3.900 y uno de cada 8.500 nacidos; en Medio Oriente, en uno de cada 2.560 a 15.876, y en Asia, en uno de cada 40.000 a 100.000 nacidos. Esta gran variabilidad depende de la consanguinidad y del origen étnico y varía mucho a nivel regional según la heterogeneidad étnica, siendo más frecuente a nivel mundial la mutación DF508 ^2^. La frecuencia de portadores sanos heterocigotos en la población caucásica se ha estimado en una de cada 25 personas ^1^^,^^3^.

En Colombia, la incidencia estimada es de uno por cada 5.000 nacidos vivos ^4^. La variabilidad en el riesgo de probabilidad de ser portador de una mutación depende del origen étnico cuando no hay antecedentes familiares de la enfermedad. En familiares del caso índice de fibrosis quística, esta probabilidad de portador de una mutación aumenta según el grado de consanguinidad.

El riesgo de ser portador de una mutación del gen regulador de conductancia transmembrana de fibrosis quística se detecta mediante pruebas genéticas que analizan de manera parcial o total los componentes del gen. Los paneles de mutación detectan las mutaciones comunes de cada región, pero ello depende de la epidemiologia genética del gen *CFTR*, ya que el análisis completo del gen es laborioso y costoso. El panel de 25 mutaciones recomendado por el *American College of Medical Genetics and Genomics* (ACMG) detecta el 83,7 % de las mutaciones del gen *CFTR*, pero dicho porcentaje difiere según las regiones ^2^.

En Colombia son pocos los estudios que contribuyen a establecer la epidemiología y la estadística de las mutaciones más frecuentes en pacientes afectados por la enfermedad y en portadores asintomáticos, ya que no es frecuente que se analice la totalidad del gen *CFTR*^1^^,^^3^^,^^4^. No existe un panel de mutaciones específico para Colombia con características operativas idóneas que pueda reducir los costos de la prueba genética.

Es importante mencionar que ni en Colombia ni en Suramérica se han hecho estudios de costo-efectividad de la prueba de secuenciación que permitan la detección de portadores de mutaciones en la fibrosis quística para la prevención de futuras concepciones con riesgo de presentar la enfermedad y esta no se está incluida en los planes de aseguramiento del sistema de salud colombiano. La guía colombiana recomienda desarrollar paneles de mutación específicos para la población colombiana que logren detectar el 80 % de los afectados y reducir el costo de los estudios moleculares ^5^.

En los padres portadores, el riesgo de transmitir ambos alelos defectuosos a hijos que desarrollen la enfermedad es del 25 % y el de tener hijos portadores sanos es del 50 %. Las opciones reproductivas para las parejas portadoras incluyen el diagnóstico prenatal con posible terminación del embarazo, la donación del oocito o de esperma, la adopción, el no tener hijos o el diagnóstico genético antes de una implantación ^6^.

El costo de un paciente con fibrosis quística se estima entre USD$ 10.000 y USD$ 40.000 por año representados en costos médicos directos y USD$ 9.000 en costos secundarios ^7^, en tanto que la prueba de secuenciación completa del gen *CFTR* tiene un costo estimado entre USD$ 1.000 y USD$ 2.000 en Colombia, por lo que constituye una posible medida costo-efectiva que debe ser evaluada e implementada en el sistema de salud colombiano.

Esta enfermedad disminuye la supervivencia a un promedio de 37 años de edad con un adecuado manejo multidisciplinario en centros especializados ^5^.

El objetivo de esta investigación fue determinar la razón de costo- efectividad de la prueba diagnóstica de secuenciación del gen *CFTR* para portadores asintomáticos en edad fértil familiares de primer, segundo y tercer grados de consanguinidad del caso índice con diagnóstico de fibrosis quística, con el fin de prevenir futuras concepciones por el riesgo de presentar fibrosis quística mediante la asesoría genética bajo la perspectiva del tercer pagador.

## Materiales y métodos

Se hizo un estudio de costo-efectividad utilizando un modelo de árbol de decisiones y un análisis de sensibilidad probabilístico. La evidencia se obtuvo a partir de una búsqueda sistemática en bases de datos especializadas en salud, en la literatura gris y con un panel de expertos en estudios de costo-efectividad y sensibilidad y especificidad de la prueba de secuenciación del *CFTR*.

La búsqueda se hizo en Medline, NHS Economic Evaluation Database, Human Genomic Epidemiology Network (HuGE Net), Embase, Cochrane Database of Systematic Reviews, LILACS, Health Technology Assessment, Genetests y Genetsickkids, sin restricción de idioma ni fecha de publicación incluyendo los términos “Cystic fibrosis, CFTR, Mucoviscidosis, Transmembrane conductance regulator gen, Sequence Analysis, Massively- Parallel Sequencing, genetic screening, carrier screening, carrier testing, Diagnostic Tests, Sensitivity, Specificity”.

Dos revisores independientes evaluaron la elegibilidad de los artículos. La evaluación de los estudios no fue cegada, ya que se conocía el nombre del autor, la institución y la fuente de publicación. Los desacuerdos fueron resueltos por un tercer revisor.

La evidencia se valoró con un instrumento de evaluación de la calidad de pruebas diagnósticas (Quadas2) y otro de evaluación de la calidad de estudios económicos en salud (QHES) ^8^^,^^9^. El diseño se hizo mediante un modelo de árbol de decisiones con el programa TreeAge Pro Healthcare 2015 tomando como unidad de análisis la prevención de futuras concepciones mediante asesoría genética por el riesgo de presentar fibrosis quística y la razón de costo- efectividad incremental. Se tuvo en cuenta la mejor ‘evidencia’ disponible para elaborar el modelo, medir, estimar y valorar los resultados, los costos directos de las pruebas diagnósticas y los costos de atención anual de un paciente con fibrosis quística, estos últimos obtenidos de los sistemas de información de la cuenta de alto costo del Ministerio de Salud y Protección Social.

Se utilizaron los costos directos reportados por las entidades promotoras de salud a la cuenta de alto costo del Ministerio de Salud y Protección Social de Colombia en el 2012. Los costos se actualizaron al valor del momento aplicando el índice de precios al consumidor en salud de Colombia para el 2013, el 2014, el 2015 y el 2016. No se tuvieron en cuenta los costos indirectos ni los costos por pérdida de productividad. Los costos de la prueba genética de secuenciación del gen *CFTR* con la técnica Sanger se obtuvieron de laboratorios genéticos nacionales y de referencias colombianas. Los valores de los costos se ajustaron a la tasa de cambio representativa del mercado (COP$ 3.382) en agosto del 2019 ^10^.

Los supuestos aplicados al modelo fueron: a) todas las parejas en riesgo por antecedentes familiares en primer, segundo o tercer grados de consanguinidad aceptarían someterse a la prueba de secuenciación del gen *CFTR*; b) el 100 % de las parejas en riesgo por tener un resultado positivo en la prueba de secuenciación aceptarían las recomendaciones dadas en la asesoría genética; c) las parejas asintomáticas portadoras de mutaciones tendrían 25 % de probabilidades de tener hijos con la enfermedad y el 50 %, hijos portadores asintomáticos de la mutación; d) las parejas analizadas recordarían su estado de portador anotado en su historia clínica y no se repetirían nuevamente sus exámenes.

Al modelo se le hizo un análisis de sensibilidad determinístico y probabilístico desde la perspectiva del tercer pagador y con un horizonte temporal a un año. Para el análisis de sensibilidad probabilístico se desarrolló una simulación de Montecarlo con 10.000 iteraciones y se utilizaron distribuciones logarítmicas normales para costos y distribuciones beta para probabilidades. Esta investigación se ajustó a la “Guía metodológica para la actualización integral del plan obligatorio de salud del Sistema General de Seguridad Social en Salud” ^11^ y a la “Guía metodológica para la elaboración de guías de atención integral en el sistema general de seguridad social en salud colombiano” ^12^.

El estudio fue clasificado como una investigación con mínimo riesgo según la Resolución 8430 de 1993, artículo 11 del Ministerio de Salud ^13^.

## Resultados

En la revisión sistemática de estudios de costo-efectividad se encontraron 573 artículos y se excluyeron 560 al revisar los títulos y los resúmenes. Se seleccionaron 13 estudios ^14^^-^^26^ que cumplían con los criterios de inclusión al ser evaluaciones económicas completas. De los seleccionados, cuatro se habían hecho en Estados Unidos, dos en el Reino Unido, tres en los Países Bajos, tres en Australia y uno en México. Seis de los estudios tenían como intervención la tamización previa a la concepción además de la tamización prenatal ^16^^,^^18^^,^^19^^,^^20^^,^^24^^,^^26^, cinco exclusivamente la tamización prenatal ^14^^,^^15^^,^^17^^,^^21^^,^^25^ y dos, la tamización previa a la concepción ^22^^,^^23^. La perspectiva más usada fue la del tercer pagador (9 de los 13 estudios incluidos). La tasa de descuento aplicada estuvo entre el 3,5 y el 5 %.

La principal unidad de análisis fue la del costo por pareja portadora detectada, seguida del costo por nacimiento con fibrosis quística evitado. Diez estudios aplicaron el modelo de análisis de decisiones. Hubo heterogeneidad en los supuestos aplicados y la mayoría utilizó el método univariado para el análisis de sensibilidad. Todos los estudios aplicaron la sensibilidad y la especificidad de la prueba de portador y de la prenatal. En todos los estudios seleccionados la intervención fue comparada con no hacer la prueba, teniendo en cuenta que la mayoría de países no habían establecido aún un programa de tamización genética para portadores de fibrosis quística. La prevalencia de portador más empleada fue de uno en 25 pacientes seguida por uno en 30, que son las de mayor aplicación en pacientes caucásicos. Se evidenció que la expectativa de vida más usada para un paciente con fibrosis quística fue de 30 años.

Se encontró una importante heterogeneidad en la metodología aplicada en los estudios, lo que desembocó en que los resultados no fueran comparables entre ellos y se concluyera que existen diferentes enfoques para el uso de esta prueba genética: características operativas de la prueba diagnóstica, moneda, y supuestos y probabilidades ajustados a las necesidades y características de cada país. No se encontró ningún estudio económico en Suramérica ^27^.

Se registró el costo total del plan obligatorio de salud (POS) y del no POS para el 2012 en pesos colombianos, el cual se actualizó al valor del 2016 aplicando el índice de precios al consumidor (IPC) en salud de Colombia: 0,0444 en el 2013; 0,0346 en el 2014; 0,053 en el 2015, y 0,0814 en el 2016. En dicho registro se encontró el costo individual y el promedio por rango de edades con una mayor frecuencia de pacientes entre los 1 y los 9 años. El costo promedio por paciente, independientemente de la edad y la gravedad de la enfermedad, fue de COP$ 35’986.538 (USD$ 10.640,60), con una desviación estándar de COP$ 21’530.047 (USD$ 6.366,06). Con esta información se determinó un costo mínimo de COP$ 14’456.491 (USD$ 4.274,53) y un costo máximo de COP$ 57’516.585 (USD$ 17.006,67). En los cuadros 1 y 2 se presentan los parámetros de costos y las probabilidades aplicadas al modelo.

**Cuadro 1:** Parámetros de costos aplicados al modelo

Variable		Costos		Fuentes	Parámetros
	Base	Mínimo	Máximo		
Costo de la prueba desecuenciación del gen CFTR	COP$ 3’474.000 USD$ 1.027,20	COP$ 3’432.904 USD$ 1.015,05	COP$ 4’234.000 USD$ 1.251,92	1. Laboratorio Genética Molecular de Colombia, 2016	Distribución log normal μ=15,0608 σ=,0559
				2. GPC FQ Colombia, 2014- 2016	
				3. Laboratorio Genetix, 2016	
Costo de la atención anual por paciente con fibrosis quística	COP$ 35’986.538 USD$ 10.640,60	COP$ 14’456.491 USD$ 4.274,53	COP$ 57’516.585 USD$ 17.006,67	1. SISPRO/2012, 2016	Distribución log normal μ=17,3986 σ=0,3452
Costo de la asesoría genética a la pareja en riesgo	COP$ 28.500 USD$ 8,42	COP$ 25.650 USD$ 7,58	COP$ 31.350 USD$ 9,26	SOAT 2016	Distribución log normal μ=10,2576 σ=0,0501

**Cuadro 2:** Probabilidades consideradas en el modelo

Variable	Base	Mínimo	Máximo	Fuentes	Parámetros
Probabilidad de ser portador de mutación para fibrosis quística con antecedente de familiar en primer grado (hermanos de pacientes con fibrosis quística)	0,66	0,25	1	Roberts, 2003 _^28^_ Expertos	Distribución beta α=4,05; β=2,08
Probabilidad de ser portador de mutación para fibrosis quística con antecedente de familiar en primer grado (hermanos de pacientes con fibrosis quística) con antecedente de familiar en segundo grado (tíos y abuelos de pacientes con fibrosis quística)	0,5	NA	NA	Roberts, 2003	NA
Probabilidad de ser portador de mutación para fibrosis quística con antecedente de familiar en tercer grado (primos de pacientes con fibrosis quística)	0,25	NA	NA	Roberts, 2003	NA
Probabilidad de ser portador de mutación para fibrosis quística sin antecedente familiar en Colombia	0,015 (1/65)	NA	NA	Keyeux, 1997 ^3^	NA
Probabilidad de ser portador de mutación para fibrosis quística sin antecedente familiar en Colombia en caucásicos	0,04 (1/25)	NA	NA	ACOG, 2011 ^29^	NA
Sensibilidad de la prueba de secuenciación del gen CFTR	0,96	0,92	0,97	Lyon, 2015 ^30^	Distribución beta α=226,59; β=9,44
Especificidad de la prueba de secuenciación del gen CFTR	1	0,99	1	Lyon, 2015 ^30^	Distribución beta α=152,48; β=0,1526

CFTR: regulador de la conductancia transmembranal de fibrosis quística

En la búsqueda sistemática de estudios sobre pruebas diagnósticas se encontró el reporte de las características operativas de la prueba de portador para fibrosis quística o de la prueba genética para pacientes con diagnóstico clínico de la enfermedad en nueve artículos, de los cuales cuatro se incluyeron en el análisis y revisión completa con el QUADAS2.

En el presente estudio se consideró que las pruebas MGL 2 y 5 evaluaban competencias diagnósticas específicas para detectar mutaciones de fibrosis quística en muestras de laboratorio purificadas con mutaciones conocidas revisadas por el comité del *College of American Pathologists*. No se establecía la selección de los pacientes, si eran portadores o no, o si habían tenido fibrosis quística. Como parte de la prueba de competencia enviada por dicho comité, se solicitaba que cada laboratorio interpretara clínicamente la mutación o las mutaciones encontradas, lo que podría definirse como un enmascaramiento simple de los laboratorios que disminuyó el riesgo de sesgos de selección y de diagnóstico ^31^.

Se encontró que el estudio de Lyon ^30^ fue el de menor riesgo de sesgo y de una alta calidad para incluirlo en el análisis, como se evidencia en el cuadro 3, ya que se reportan de manera diferencial las características analíticas de laboratorios internacionales no estadounidenses y las técnicas de diagnóstico aplicadas a las muestras, entre ellas la de secuenciación de Sanger, aunque solo se incluyeron en las muestras enviadas las 23 mutaciones más frecuentes recomendadas por el panel de detección del *American College of Obstetricians and Gynecologists* (ACOG) y del ACMG.

**Cuadro 3:** Resultados del *Quality Assessment of Diagnostic Accuracy Studies* (QUADAS 2)

Estudio	Riesgo de sesgo				Aplicabilidad		
	Selección de pacientes	Prueba índice	Prueba de referencia	Flujo y cronograma	Selección de pacientes	Prueba índice	Prueba de referencia
Palomaki, 2003 ^33^	?	?	☺	☺	?	?	☺
Strom, 2003 ^34^	☺	☺	?	?	☺	☺	?
Pratt, 2009 ^35^	?	☺	?	?	?	☺	?
Lyon, 2015	?	☺	☺	☺	☺	☺	☺

☺ Bajo riesgo ☺ Alto riesgo ? Riesgo poco claro

**Figura 1.**
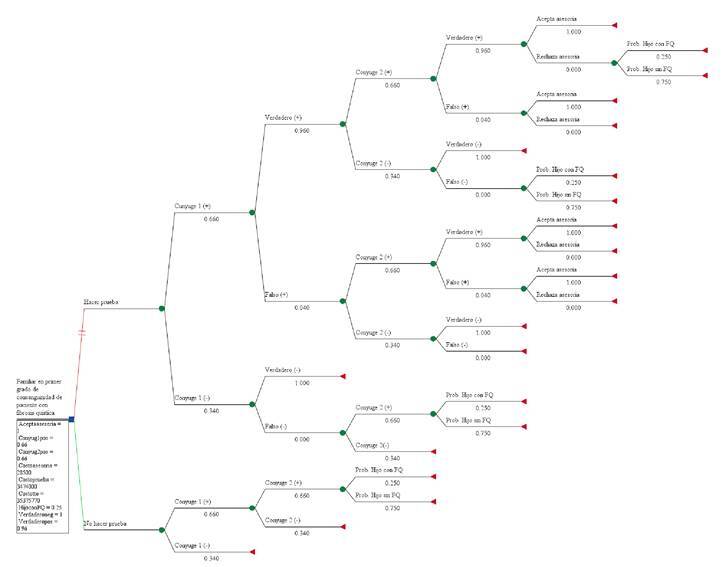
Árbol de decisiones aplicado al modelo

Al hacer el análisis de costo-efectividad con el modelo de árbol de decisiones (figura 1), se obtuvo una RICE de COP$ 17’082.833,90 (USD$ 5.051,10), lo que evidencia que este es el costo incremental por obtener 10,89 % más de probabilidades de evitar el nacimiento de un niño enfermo con fibrosis quística por pareja tamizada, como se aprecia en tabla 4. La estrategia de no hacer la prueba genética tuvo una efectividad basal de 89,11 % para tener niños sanos por cada gestación con base en las características de herencia autosómica recesiva propias de la enfermedad. Con la prueba genética acompañada de asesoría, se logró obtener el 100 % de efectividad para evitar el nacimiento de niños con fibrosis quística.

Conyuge (+): portador de mutación; conyuge (-): no portador de mutación; verdadero (+) (-)/falso (+)(-): correspondiente a las caracteristicas operativas de la prueba según sensibilidad y especificidad; Pro. hijo con o sin fibrosis quística: probabilidad de tener o no un hijo con fibrosis quistica; rechaza o acepta asesoría: asesoría genética sobre el resultado de la prueba con sus recomendaciones

**Cuadro 4:** Resultado del análisis de costo-efectividad

Estrategia	Costo	Costo incremental	Efectividad	Efectividad incremental	ICER
No hacer la prueba	COP$ 3’918.933,99 USD$ 1.158,76	NA	0,8911	NA	NA
Hacer la prueba	COP$ 5’779.254,60 USD$ 1.708,82	COP$ 1’860.320,61 USD$ 550,06	1	0,1089	COP$ 17’082.833,90 USD$ 5.051,10

De este análisis se pudo inferir que de cada 100 gestaciones de parejas en riesgo que no se hicieran la prueba genética, 89 nacimientos serían de niños sanos y 11 estarían afectados con fibrosis quística. Con la estrategia propuesta, en cada 100 parejas en riesgo sometidas a la prueba y con asesoría genética, se evitarían 11 concepciones de niños con fibrosis quística. En el plano del costo-efectividad se encontró que las estrategias se situaban en el cuadrante I, lo que evidenció que hacer la prueba genética era más efectivo, pero también más costoso que no hacerla. Para los familiares de segundo y tercer grados de consanguinidad se encontró una RICE de COP$ 65’546.360,2 (USD$ 19.380,94) y COP$ 189’099.579,5 (USD$ 55.913,53), respectivamente.

**Figura 2:**
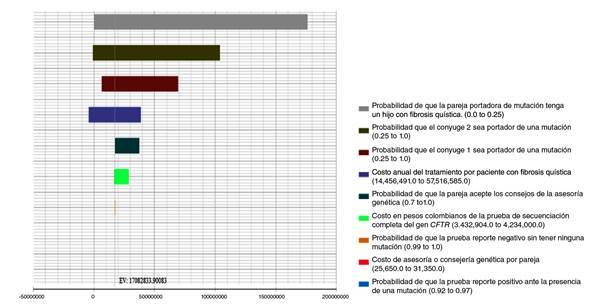
Diagrama de tornado (ICER)

 En el análisis de sensibilidad univariado representado en el diagrama de tornado(figure 2), se encontró que las variables con mayores probabilidades de riesgo de incertidumbre fueron: hijo con fibrosis quística, ambos cónyuges positivos (hombre y mujer) y costo del tratamiento, lo cual indica que el cambio de cada una de estas variables de manera independiente entre los rangos establecidos influyó directamente en el resultado, siendo menor o mayor a la RICE obtenida con los datos basales del modelo. En la variable de un hijo con fibrosis quística, el riesgo de tener hijos con fibrosis quística cuando ambos padres son portadores de una mutación fue de hasta el 25 %, por ser esta una enfermedad autosómica recesiva. En cuanto a las variables de costo de tratamiento y uno de los cónyuges positivo, se encontró que la RICE llega a ser negativa, lo que representa un ahorro con respecto a los límites superiores de dichas variables. El resto de las variables no presentó una proporción de incertidumbre considerable.

Se hizo un análisis bivariado de la variable “probabilidad de portador de mutación” en ambos cónyuges y se encontró una RICE mucho más alta comparada con los parámetros de base: aproximadamente, entre COP$ 65.000.000 (USD$ 19.219,39) y COP$ 189’000.000 (USD$ 55.884,09) por evitar una futura concepción de un niño con riesgo de presentar fibrosis quística.

Teniendo en cuenta que el presente estudio no fue de costo-utilidad y respondió a la perspectiva del tercer pagador, se usó el producto interno bruto (PIB) per cápita como valor de disponibilidad a pagar sin aplicar los años de vida con discapacidad (AVAD) como unidades de análisis. Según la comisión de macroeconomía de la Organización Mundial de la Salud (OMS) y la Organización Panamericana de la Salud (OPS), el PIB per cápita se puede aplicar para medir el costo-efectividad de una tecnología en salud para enfermedades comunes ^32^. Se aplicó esta consideración al caso de la fibrosis quística, ya que no existe un umbral nacional de disponibilidad a pagar para la prevención de esta enfermedad, clasificada como huérfana de alto costo. Si la RICE es menor o igual a un PIB per cápita, se considera muy costo-efectiva, entre uno y tres PIB per cápita se considera costo-efectiva y más de tres PIB per cápita como no costo-efectiva. Según estimaciones del DANE, el PIB per cápita provisional para el 2015 fue de COP$ 16’613.951 (USD$ 4.912,46) ^10^.

Con la simulación de Monte Carlo se encontró que esta tecnología diagnóstica fue costo-efectiva por tener una RICE entre 1 y 3 PIB per cápita considerando la incertidumbre bajo un análisis de sensibilidad probabilístico. La probabilidad de que la estrategia de hacer la prueba diagnóstica fuera costo-efectiva fue de 39 %, 61,18 % y 74,36 % con uno, dos y tres PIB per cápita, respectivamente.

**Figura 3:**
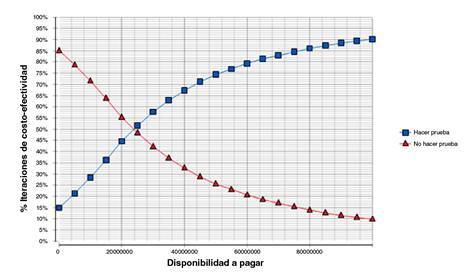
Curva de aceptabilidad de costo-efectividad

La curva de aceptabilidad de costo-efectividad (figure 3) evidenció que con una disponibilidad a pagar de COP$ 25’000.000 (USD$ 7.392,07) la probabilidad de que la estrategia de hacer la prueba genética fuera costo- efectiva era del 50 %. El resultado de la RICE de COP$ 17’082.833,90 (USD$ 5.051,10) evidenció que la probabilidad de que la estrategia de hacer la prueba genética fuera costo-efectiva era del 36 %, aproximadamente. Además, se evidenció que la probabilidad de costo-efectividad de hacer la prueba genética con una RICE del 60 % estaba en un rango de disponibilidad a pagar de COP$ 4’000.000 (USD$ 1.182,73) y COP$ 60’000.000 (USD$ 17.740,98. 

## Discusión

Los datos demuestran que puede existir variabilidad entre la aceptación o no de la prueba genética para los portadores de fibrosis quística y de las recomendaciones dadas durante la asesoría genética que impliquen opciones como la de evitar tener hijos o adoptar, lo que no representaría costos adicionales. Otras opciones reproductivas, como el diagnóstico prenatal, la donación de oocitos o de esperma y el diagnóstico genético antes de la implantación sí tienen un costo adicional que no se contempló en el modelo, pues la pareja tiene autonomía para decidir sobre estas alternativas y son quienes optan entre abortar o no en caso de que el neonato esté afectado con la enfermedad.

Es importante tener en cuenta que este estudio se planteó en circunstancias ideales en las que el 100 % de las parejas aceptaría usar la prueba de portador para fibrosis quística y las recomendaciones de la asesoría genética. Dado este supuesto, se evaluó el resultado dependiendo de la probabilidad de aceptación de las recomendaciones dadas durante la asesoría genética y demostrando que con el 10 % de aceptación de la asesoría genética por cada 100 parejas en riesgo de ser portadoras de la mutación se evitaría, por lo menos, un caso de fibrosis quística. Asimismo, se determinó el costo total de los 11 casos de fibrosis quística por cada 100 parejas en riesgo en caso de no hacerse la prueba, con un total de COP$ 13.315’019.060 (USD$ 3’937.025,15), según la esperanza de vida promedio de 37 años en Colombia y asumiendo el costo anual promedio como constante.

Ioannou, *et al*. ^36^, hicieron en el 2014 una revisión sistemática de estudios que evaluaban las consecuencias psicológicas y de preferencia en cuanto al uso de la prueba en familiares con antecedentes de fibrosis quística. Se seleccionaron 85 estudios en los que se había registrado el 40 % de aplicación de la prueba prenatal y el 30,6 % antes de la concepción. El 89 % de las mujeres no gestantes y el 98 % de las gestantes prefería que la prueba se ofreciera antes de la concepción. El rango de su uso en el seguimiento antes de la concepción fue entre 2 y 96 %. En 50 a 94 % de los estudios se encontró que la población prefería recibir asesoría antes de la prueba en la consulta general.

En su estudio del 2015, Ioannou, *et al.*^37^, analizaron ocho parejas de un programa de tamización para portadores del gen de la fibrosis quística, seis de las cuales decidieron hacerse el diagnóstico prenatal y se encontraron dos fetos afectados por la enfermedad. Tres parejas decidieron no tener más hijos.

Por su parte, en un estudio retrospectivo (25 años) del 2016 en Francia, Dugueperoux, *et al.*^38^, analizaron la probabilidad del uso de la prueba de portador de mutaciones para fibrosis quística y encontraron que la habían usado el 40,3 % (185 personas) de las 459 personas mayores de 18 años familiares de 40 pacientes con fibrosis quística. Esta estrategia permitió la detección de cinco parejas portadoras con riesgo de tener hijos con la enfermedad.

Como todo modelo, el aquí empleado también tuvo limitaciones:

- no se valoró desde la perspectiva social y no se tuvieron en cuenta los costos indirectos;
- al ser un modelo con un horizonte temporal de un año, no se pudieron evaluar resultados posteriores, como el tener más de una gestación, o considerar la esperanza de vida asociada con los costos de atención en salud, con lo cual mejoraría el costo-efectividad;
- las probabilidades aplicadas en el modelo se obtuvieron de estudios internacionales que evaluaban las características operativas de la prueba genética en poblaciones principalmente estadounidenses y europeas, lo que limita su valoración en la población colombiana;
- no se ha establecido un umbral de disponibilidad de pagar para la prevención de casos de fibrosis quística en Colombia, lo cual deja su determinación para la prevención de un caso de fibrosis quística a consideración del sistema de salud y del tomador de decisiones, lo que podría variar la clasificación del costo-efectividad de tecnologías en salud para enfermedades de alto costo; asimismo, el umbral de disponibilidad a pagar para la prevención de casos de fibrosis quística asociado con la esperanza de vida de la enfermedad podría mejorar el resultado de costo-efectividad del modelo;
- se tuvo en cuenta que el costo de la prueba fue el de la secuenciación completa del gen *CFTR*, ya que no se encontraron estudios epidemiológicos de la distribución genética completa de las mutaciones del gen de la fibrosis quística en el país, lo que habría permitido aplicar un panel de mutaciones con alta sensibilidad y especificidad para la población colombiana; si se contara con este tipo de panel de mutaciones específico para Colombia, los costos de la prueba genética bajarían y ello mejoraría su costo-efectividad, y
- el PIB per cápita como umbral de disponibilidad de pagar pudo sobrevalorar o subvalorar el costo-efectividad del modelo.

En conclusión, se evidenció que la prueba genética de secuenciación para portadores de fibrosis quística es costo-efectiva según la disponibilidad de pagar de 1 a 3 PIB per cápita por parte del tercer pagador. Para los familiares en segundo y tercer grado de consanguinidad el ICER es mayor y el costo-efectividad depende de la disponibilidad de pagar. No se encontraron estudios sobre el costo-efectividad de esta prueba genética en portadores de fibrosis quística en Colombia ni en Suramérica y tampoco se ha establecido un umbral de disponibilidad de pagar para la prevención de casos en enfermedades de alto costo, como la fibrosis quística.

Deben hacerse más estudios de distribución epidemiológica de las mutaciones más frecuentes de fibrosis quística en pacientes afectados y familiares portadores para establecer un panel de mutaciones con alta sensibilidad y especificidad para la población colombiana. Dado el costo de la prueba genética y el amplio número de mutaciones que aparecen en el gen que regula la fibrosis quística, es importante crear un panel de mutaciones específico para reducir los costos de implementación de la prueba y así elevar el costo-efectividad según lo evidenciado en los resultados y en el costo anual de un paciente con fibrosis quística, sin considerar las complicaciones y los años vividos con discapacidad que esta enfermedad genera. Resultaría muy útil implementar esta prueba genética en la población colombiana con un costo bajo para garantizar una mejor distribución de los recursos en salud según la carga de la enfermedad a nivel nacional.

## References

[1] Jay ML, Mateus H, Fonseca D, Restrepo MC, Keyeux G (2006). PCR- heteroduplex por agrupamiento: implementación de un método de identificación de portadores de la mutación más común causal de fibrosis quística en Colombia. Colomb Med.

[2] World Health Organization (1983). The molecular genetic epidemiology of cystic fibrosis.

[3] Keyeux G, Sánchez D, Garavito P, Stand I, Rodas C, Bienvenu T (1997). Estudios moleculares en pacientes colombianos con fibrosis quística. Acta Médica Colombiana.

[4] Silva MS (2007). Determinación de la frecuencia de portadores de la mutación DF508 en la población colombiana.

[5] Ministerio de Salud y Protección Social (2014). Colciencias. Guía de práctica clínica para la prevención, diagnóstico, tratamiento y rehabilitación de fibrosis quística.

[6] Goetzinger KR, Cahill AG (2010). An update on cystic fibrosis screening. Clin Lab Med.

[7] National Institutes of Health (1999). Consensus Development Conference Statement on Genetic Testing for Cystic Fibrosis. Genetic testing for cystic fibrosis. Arch Intern Med.

[8] Whiting PF, Rutjes AW, Westwood ME, Mallett S, Deeks JJ, Reitsma JB (2011). QUADAS-2: A revised tool for the quality assessment of diagnostic accuracy studies. Ann Intern Med.

[9] Chiou CF, Hay JW, Wallace JF, Bloom BS, Neumann PJ, Sullivan SD (2003). Development and validation of a grading system for the quality of cost-effectiveness studies. Med Care.

[10] Banco de la República de Colombia Dirección de Síntesis y Cuentas Nacionales, estudios económicos - Cuentas financieras.

[11] Comisión de Regulación en Salud (201). Metodología para la actualización integral del plan obligatorio de salud del sistema general de seguridad social en salud.

[12] Ministerio de la Protección Social, Colciencias (2010). Centro de Estudios e Investigación en Salud de la Fundación Santa Fe de Bogotá, Escuela de Salud Pública de la Universidad de Harvard. Guía metodológica para el desarrollo de guías de atención integral en el Sistema General de Seguridad Social en Salud colombiano.

[13] Ministerio de Salud Resolución 8430 de 1993.

[14] Lieu TA, Watson SE, Washington AE (1994). The cost-effectiveness of prenatal carrier screening for cystic fibrosis. Obstet Gynecol.

[15] Asch DA, Hershey JC, Dekay ML, Pauly MV, Patton JP, Jedrziewski MK (1998). Carrier screening for cystic fibrosis: Costs and clinical outcomes. Med Decis Making.

[16] Morris JK, Oppenheimer PM (1995). Cost comparison of different methods of screening for cystic fibrosis. J Med Screen.

[17] Cuckle HS, Richardson GA, Sheldon TA, Quirke P (1995). Cost effectiveness of antenatal screening for cystic fibrosis. BMJ.

[18] van der Riet AA, van Hout BA, Rutten FF (1997). Cost effectiveness of DNA diagnosis for four monogenic diseases. J Med Genet.

[19] Wildhagen MF, Hilderink HB, Verzijl JG, Verheij JB, Kooij L, Tijmstra T (1998). Costs, effects, and savings of screening for cystic fibrosis gene carriers. J Epidemiol Community Health.

[20] Rowley PT, Loader S, Kaplan RM (1998). Prenatal screening for cystic fibrosis carriers: An economic evaluation. Am J Hum Genet.

[21] Doyle NM, Gardner MO (2003). Prenatal cystic fibrosis screening in Mexican Americans: An economic analysis. Am J Obstet Gynecol.

[22] Warren E, Anderson R, Proos AL, Burnett LB, Barlow-Stewart K, Hall J (2005). Cost-effectiveness of a school-based Tay-Sachs and cystic fibrosis genetic carrier screening program. Genet Med.

[23] Weijers-Poppelaars FA, Wildhagen MF, Henneman L, Cornel MC, Kate LP (2005). Preconception cystic fibrosis carrier screening costs and consequences. Genet Test.

[24] Wei S, Quigg MH, Monaghan KG (2007). Is cystic fibrosis carrier screening cost effective?. Community Genet.

[25] Maxwell S, Brameld K, Youngs L, Geelhoed E, O’leary P (2010). Informing policy for the Australian context - Costs, outcomes and cost savings of prenatal carrier screening for cystic fibrosis. Aust N Z J Obstet Gynaecol.

[26] Norman R, van Gool K, Hall J, Delatycki M, Massie J (2012). Cost-effectiveness of carrier screening for cystic fibrosis in Australia. J Cyst Fibros.

[27] Andrade E, Díaz JA (2016). Revisión sistemática de estudios de costo-efectividad del test de portadores para fibrosis quística. Revista de la Facultad de Medicina.

[28] Roberts T, Schwarz MJ, Kerr-Liddell R, Hinks JL, Super M (2003). Cascade carrier-testing in cystic fibrosis. Paediatr Respir Rev.

[29] American College of Obstetricians and Gynecologists (2011). Update on carrier screening for cystic fibrosis. Committee Opinion No 486. Obstet Gynecol.

[30] Lyon E, Schrijver I, Weck KE, Gonzalez A, Richards CS (2015). Palomaki GE. Molecular genetic testing for cystic fibrosis: Laboratory performance on the College of American Pathologists external proficiency surveys. Genet Med.

[31] Andrade E, Díaz JA (2017). Evaluación de la costo-efectividad de la prueba de secuenciación completa del gen *CFTR* por técnica Sanger para portadores asintomáticos en población colombiana de primer, segundo y tercer grado de consanguinidad con historia familiar de fibrosis quística.

[32] WHO Commission on Macroeconomics and Health (2001). Macroeconomics and health: Investing in health for economic development / report of the Commission on Macroeconomics and Health.

[33] Palomaki EG, Bradley AL, Richards SC, Haddow EJ (2003). Analytic validity of cystic fibrosis testing: A preliminary estimate. Genet Med.

[34] Strom MC, Huang D, Chen C, Buller A, Peng M, Quan F (2003). Extensive sequencing of the cystic fibrosis transmembrane regulator gene: Assay validation and unexpected benefits of developing a comprehensive test. Genet Med.

[35] Pratt M. V, Caggana M, Bridges C, Buller MA, DiAntonio L, Highsmith EW (2009). Development of genomic reference materials for cystic fibrosis genetic testing. J Mol Diagn.

[36] Ioannou L, McClaren BJ, Massie J, Lewis S, Metcalfe SA, Forrest L (2014). Population-based carrier screening for cystic fibrosis: A systematic review of 23 years of research. Genet Med.

[37] Ioannou L, Delatycki MB, Massie J, Hodgson J, Lewis S (2015). “Suddenly having two positive people who are carriers is a whole new thing”- Experiences of couples both identified as carriers of cystic fibrosis through a population-based carrier screening program in Australia. J Genet Couns.

[38] Duguépéroux I, L’Hostis C, Audrézet MP, Rault G, Frachon I, Bernard R (2016). Highlighting the impact of cascade carrier testing in cystic fibrosis families. J Cyst Fibros.

